# Supplementary material for: A fully automated high-throughput workflow for 3D-based chemical screening in human midbrain organoids
Source: eLife. 2020 Nov 3;9:e52904. doi: 10.7554/eLife.52904 (PMC7609049; doi:10.7554/eLife.52904)
Supplement: Supplementary file 1. [file elife-52904-supp1.docx]

**Supplementary file 1. Source data for the calculation of sample retention efficiency shown in Figure 1d**

The data here is shown as % sample retention for each step per 96-well plate, each value in the tables below represents one individual plate.

**Step 1 – Sample retention after 30 days of automated culture**

| 100.00% | 100.00% | 100.00% |
| --- | --- | --- |
| 98.96% | 98.96% | 100.00% |
| 100.00% | 100.00% | 100.00% |
| 98.96% | 100.00% | 100.00% |
| 100.00% | 100.00% | 97.92% |
| 100.00% | 100.00% | 100.00% |
| 100.00% | 98.96% | 100.00% |
| 100.00% | 100.00% | 100.00% |
| 97.92% | 100.00% | 100.00% |
| 100.00% | 100.00% | 97.92% |

**Step 2 – Efficiency of the automated transfer from 96-well V-bottom culture to imaging plates**

| 97.92% | 93.75% | 96.34% |
| --- | --- | --- |
| 91.86% | 98.96% | 100.00% |

**Step 3 – Percentage of samples acquired by the automated high content confocal imaging system that pass image analysis quality control**

| 93.75% | 92.71% | 95.31% |
| --- | --- | --- |
