# Supplementary material for: A fully automated high-throughput workflow for 3D-based chemical screening in human midbrain organoids
Source: eLife. 2020 Nov 3;9:e52904. doi: 10.7554/eLife.52904 (PMC7609049; doi:10.7554/eLife.52904)
Supplement: Supplementary file 2. [file elife-52904-supp2.docx]

**Supplementary file 2. Complete List of gene ontology (GO) terms for genes significantly (p < 0.001) upregulated (log2 fold change > 2) in AMOs compared to published midbrain organoids (Jo et al., 2016)**

| **GO Term** | **Description** | **P-value** |
| --- | --- | --- |
| GO:0099536 | synaptic signaling | 2.82E-16 |
| GO:0099537 | trans-synaptic signaling | 2.82E-16 |
| GO:0098916 | anterograde trans-synaptic signaling | 1.78E-15 |
| GO:0007268 | chemical synaptic transmission | 1.78E-15 |
| GO:0007610 | behavior | 2.03E-15 |
| GO:0007267 | cell-cell signaling | 5.60E-15 |
| GO:0023052 | signaling | 6.55E-15 |
| GO:0050877 | nervous system process | 6.90E-14 |
| GO:0050890 | cognition | 3.44E-13 |
| GO:0007154 | cell communication | 7.75E-12 |
| GO:0007611 | learning or memory | 1.15E-11 |
| GO:0042391 | regulation of membrane potential | 6.23E-10 |
| GO:0050804 | modulation of chemical synaptic transmission | 6.75E-10 |
| GO:0099177 | regulation of trans-synaptic signaling | 7.24E-10 |
| GO:0003008 | system process | 1.81E-09 |
| GO:0043269 | regulation of ion transport | 1.37E-08 |
| GO:0007612 | learning | 1.47E-08 |
| GO:0030001 | metal ion transport | 1.63E-08 |
| GO:0060078 | regulation of postsynaptic membrane potential | 3.39E-08 |
| GO:0044057 | regulation of system process | 6.75E-08 |
| GO:0034765 | regulation of ion transmembrane transport | 7.50E-08 |
| GO:0006812 | cation transport | 1.34E-07 |
| GO:0007613 | memory | 1.36E-07 |
| GO:0006813 | potassium ion transport | 1.74E-07 |
| GO:0051966 | regulation of synaptic transmission, glutamatergic | 2.94E-07 |
| GO:0034762 | regulation of transmembrane transport | 3.70E-07 |
| GO:0051049 | regulation of transport | 4.38E-07 |
| GO:0007155 | cell adhesion | 4.89E-07 |
| GO:0022610 | biological adhesion | 5.85E-07 |
| GO:0006811 | ion transport | 6.10E-07 |
| GO:0048167 | regulation of synaptic plasticity | 7.81E-07 |
| GO:0031644 | regulation of neurological system process | 8.47E-07 |
| GO:0032501 | multicellular organismal process | 1.11E-06 |
| GO:0034220 | ion transmembrane transport | 1.77E-06 |
| GO:0007626 | locomotory behavior | 2.02E-06 |
| GO:0007600 | sensory perception | 2.08E-06 |
| GO:0050808 | synapse organization | 2.20E-06 |
| GO:0051239 | regulation of multicellular organismal process | 2.20E-06 |
| GO:0051960 | regulation of nervous system development | 2.24E-06 |
| GO:0032879 | regulation of localization | 2.46E-06 |
| GO:0048856 | anatomical structure development | 2.47E-06 |
| GO:0050806 | positive regulation of synaptic transmission | 3.04E-06 |
| GO:0098655 | cation transmembrane transport | 4.16E-06 |
| GO:0050905 | neuromuscular process | 4.27E-06 |
| GO:0051962 | positive regulation of nervous system development | 5.93E-06 |
| GO:0015672 | monovalent inorganic cation transport | 6.75E-06 |
| GO:0045664 | regulation of neuron differentiation | 1.34E-05 |
| GO:0035418 | protein localization to synapse | 2.32E-05 |
| GO:0019233 | sensory perception of pain | 2.63E-05 |
| GO:0030534 | adult behavior | 2.84E-05 |
| GO:0007158 | neuron cell-cell adhesion | 2.89E-05 |
| GO:1903522 | regulation of blood circulation | 3.06E-05 |
| GO:0008016 | regulation of heart contraction | 3.14E-05 |
| GO:0007215 | glutamate receptor signaling pathway | 3.45E-05 |
| GO:0098660 | inorganic ion transmembrane transport | 3.91E-05 |
| GO:0045666 | positive regulation of neuron differentiation | 4.01E-05 |
| GO:0071625 | vocalization behavior | 4.11E-05 |
| GO:0007186 | G protein-coupled receptor signaling pathway | 4.41E-05 |
| GO:0050767 | regulation of neurogenesis | 4.53E-05 |
| GO:0010975 | regulation of neuron projection development | 4.66E-05 |
| GO:0019932 | second-messenger-mediated signaling | 4.67E-05 |
| GO:0051967 | negative regulation of synaptic transmission, glutamatergic | 5.23E-05 |
| GO:0098742 | cell-cell adhesion via plasma-membrane adhesion molecules | 5.93E-05 |
| GO:0050805 | negative regulation of synaptic transmission | 6.47E-05 |
| GO:0021615 | glossopharyngeal nerve morphogenesis | 6.97E-05 |
| GO:0050807 | regulation of synapse organization | 7.55E-05 |
| GO:0007156 | homophilic cell adhesion via plasma membrane adhesion molecules | 7.84E-05 |
| GO:2000344 | positive regulation of acrosome reaction | 8.54E-05 |
| GO:0098662 | inorganic cation transmembrane transport | 1.08E-04 |
| GO:2000026 | regulation of multicellular organismal development | 1.08E-04 |
| GO:0019722 | calcium-mediated signaling | 1.08E-04 |
| GO:0021602 | cranial nerve morphogenesis | 1.32E-04 |
| GO:0007420 | brain development | 1.45E-04 |
| GO:0048839 | inner ear development | 1.53E-04 |
| GO:0007187 | G protein-coupled receptor signaling pathway, coupled to cyclic nucleotide second messenger | 1.59E-04 |
| GO:0051240 | positive regulation of multicellular organismal process | 1.73E-04 |
| GO:0007632 | visual behavior | 1.77E-04 |
| GO:1905516 | positive regulation of fertilization | 1.93E-04 |
| GO:0032412 | regulation of ion transmembrane transporter activity | 1.94E-04 |
| GO:0035176 | social behavior | 2.03E-04 |
| GO:0051703 | intraspecies interaction between organisms | 2.03E-04 |
| GO:0050769 | positive regulation of neurogenesis | 2.20E-04 |
| GO:0051963 | regulation of synapse assembly | 2.23E-04 |
| GO:0055085 | transmembrane transport | 2.38E-04 |
| GO:0008038 | neuron recognition | 2.63E-04 |
| GO:0097553 | calcium ion transmembrane import into cytosol | 2.65E-04 |
| GO:0048169 | regulation of long-term neuronal synaptic plasticity | 2.72E-04 |
| GO:2001028 | positive regulation of endothelial cell chemotaxis | 2.73E-04 |
| GO:0022898 | regulation of transmembrane transporter activity | 2.85E-04 |
| GO:0098693 | regulation of synaptic vesicle cycle | 2.86E-04 |
| GO:0050770 | regulation of axonogenesis | 2.93E-04 |
| GO:0098609 | cell-cell adhesion | 2.97E-04 |
| GO:2001257 | regulation of cation channel activity | 3.13E-04 |
| GO:0021546 | rhombomere development | 3.35E-04 |
| GO:0060284 | regulation of cell development | 3.48E-04 |
| GO:0010959 | regulation of metal ion transport | 4.47E-04 |
| GO:0060402 | calcium ion transport into cytosol | 4.88E-04 |
| GO:0060046 | regulation of acrosome reaction | 5.01E-04 |
| GO:0071805 | potassium ion transmembrane transport | 5.16E-04 |
| GO:0071804 | cellular potassium ion transport | 5.16E-04 |
| GO:0032502 | developmental process | 5.31E-04 |
| GO:0032409 | regulation of transporter activity | 5.51E-04 |
| GO:0099505 | regulation of presynaptic membrane potential | 5.75E-04 |
| GO:0098976 | excitatory chemical synaptic transmission | 5.75E-04 |
| GO:0007270 | neuron-neuron synaptic transmission | 5.75E-04 |
| GO:0006937 | regulation of muscle contraction | 5.76E-04 |
| GO:1905606 | regulation of presynapse assembly | 5.99E-04 |
| GO:0060079 | excitatory postsynaptic potential | 6.11E-04 |
| GO:0051969 | regulation of transmission of nerve impulse | 6.54E-04 |
| GO:0071294 | cellular response to zinc ion | 6.54E-04 |
| GO:0021599 | abducens nerve formation | 6.82E-04 |
| GO:0035284 | brain segmentation | 6.82E-04 |
| GO:0060012 | synaptic transmission, glycinergic | 6.82E-04 |
| GO:0099174 | regulation of presynapse organization | 7.14E-04 |
| GO:0010720 | positive regulation of cell development | 7.20E-04 |
| GO:0007188 | adenylate cyclase-modulating G protein-coupled receptor signaling pathway | 7.92E-04 |
| GO:0006816 | calcium ion transport | 8.08E-04 |
| GO:0006836 | neurotransmitter transport | 8.14E-04 |
| GO:0051480 | regulation of cytosolic calcium ion concentration | 8.81E-04 |
| GO:0097091 | synaptic vesicle clustering | 9.02E-04 |
| GO:0021603 | cranial nerve formation | 9.02E-04 |
| GO:0030182 | neuron differentiation | 9.80E-04 |
| GO:0009581 | detection of external stimulus | 9.85E-04 |
| GO:0023061 | signal release | 9.96E-04 |
