# Supplementary material for: A fully automated high-throughput workflow for 3D-based chemical screening in human midbrain organoids
Source: eLife. 2020 Nov 3;9:e52904. doi: 10.7554/eLife.52904 (PMC7609049; doi:10.7554/eLife.52904)
Supplement: Supplementary file 3. [file elife-52904-supp3.docx]

**Supplementary file 3. List of primary antibodies in this study**

| **Antigen** | **Species** | **Dilution** | **Supplier** | **Ref.-No.** |
| --- | --- | --- | --- | --- |
|  |  |  |  |  |
| Brn2 | Rabbit | 1 in 2000 | Cell Signaling | 12137 |
|  |  |  |  |  |
| Cleaved Caspase-3 | Rabbit | 1 in 100 | Cell Signaling | 9664 |
|  |  |  |  |  |
| Ctip2 | Rat | 1 in 750 | Abcam | ab18465 |
|  |  |  |  |  |
| DCX | Goat | 1 in 500 | Santa Cruz | sc-8066 |
|  |  |  |  |  |
| FoxA2 | Mouse | 1 in 100 | Santa Cruz | sc-101060 |
|  |  |  |  |  |
| FoxG1 | Rabbit | 1 in 500 | Abcam | ab18259 |
|  |  |  |  |  |
| GFAP | Chicken | 1 in 500 | Merck Millipore | AB5541 |
|  |  |  |  |  |
| Lmx1a | Rabbit | 1 in 100 | Abcam | ab139726 |
|  |  |  |  |  |
| Homer | Mouse | 1 in 250 | Synaptic Systems | 160 011 |
|  |  |  |  |  |
| Map2 | Chicken | 1 in 500 | Abcam | ab5392 |
|  |  |  |  |  |
| Map2 | Mouse | 1 in 1000 | Merck Millipore | MAB3418 |
|  |  |  |  |  |
| Map2 | Rabbit | 1 in 500 | Abcam | ab32454 |
|  |  |  |  |  |
| Nestin | Mouse | 1 in 250 | Life Technologies | MA1-110 |
|  |  |  |  |  |
| Nurr1 | Mouse | 1 in 100 | Santa Cruz | sc-376984 |
|  |  |  |  |  |
| Pax6 | Rabbit | 1 in 500 | BioLegend | 901301 |
|  |  |  |  |  |
| Pitx3 | Rabbit | 1 in 100 | Merck Millipore | AB5722 |
|  |  |  |  |  |
| S100b | Rabbit | 1 in 500 | Dako | Z031129-2 |
|  |  |  |  |  |
| Satb2 | Mouse | 1 in 500 | Abcam | ab51502 |
|  |  |  |  |  |
| Sox2 | Goat | 1 in 200 | R&D Systems | AF2018 |
|  |  |  |  |  |
| Synapsin1 | Mouse | 1 in 1000 | Synaptic Systems | 106 001 |
|  |  |  |  |  |
| Synaptophysin1 | Rabbit | 1 in 200 | Synaptic Systems | 101 002 |
|  |  |  |  |  |
| Tbr1 | Rabbit | 1 in 500 | Abcam | ab31940 |
|  |  |  |  |  |
| Tbr2 | Chicken | 1 in 500 | Merck Millipore | AB15894 |
|  |  |  |  |  |
| TUBB3 | Mouse | 1 in 500 | BioLegend | 801202 |
|  |  |  |  |  |
| TH | Chicken | 1 in 1000 | Abcam | ab76442 |
|  |  |  |  |  |
| TH | Rabbit | 1 in 500 | Abcam | ab112 |
|  |  |  |  |  |
| vGAT | Mouse | 1 in 100 | Synaptic Systems | 131 011 |
|  |  |  |  |  |
| vGLUT1 | Rabbit | 1 in 100 | Synaptic Systems | 13 5303 |
