# Supplementary material for: A fully automated high-throughput workflow for 3D-based chemical screening in human midbrain organoids
Source: eLife. 2020 Nov 3;9:e52904. doi: 10.7554/eLife.52904 (PMC7609049; doi:10.7554/eLife.52904)
Supplement: Supplementary file 4. [file elife-52904-supp4.docx]

**Supplementary file 4. List of quantitative real-time PCR primers in this study**

| **Gene** | **Forward primer (5’-3’)** | **Reverse primer (5’-3’)** |
| --- | --- | --- |
|  |  |  |
| AADC | TGCGAGCAGAGAGGGAGTAG | TGAGTTCCATGAAGGCAGGATG |
|  |  |  |
| Brn2 | CGGCGGATCAAACTGGGATTT | TTGCGCTGCGATCTTGTCTAT |
|  |  |  |
| DCX | AGGGCTTTCTTGGGTCAGAGG | GCTGCGAATCTTCAGCACTCA |
|  |  |  |
| EN1 | CCCTGGTTTCTCTGGGACTT | GCAGTCTGTGGGGTCGTATT |
|  |  |  |
| GAPDH | CTGGTAAAGTGGATATTGTTGCCAT | TGGAATCATATTGGAACATGTAAACC |
|  |  |  |
| GIRK2 | ATGGATCAGGACGTCGAAAG | CGGTCAGGTAGCGATAGGTC |
|  |  |  |
| GLAST | CCAGCAGGGAGTCCGTAAAC | GCAGCACAAAAGCATTCCGA |
|  |  |  |
| LMX1B | ATCGTGGCCATGGAACAGAG | GTCTGAGGAGCCGAGGAAG |
|  |  |  |
| MAP2 | CTCAGCACCGCTAACAGAGG | CATTGGCGCTTCGGACAAG |
|  |  |  |
| MBP | ATCCAAGTACCTGGCCACAG | CAAGGATGCCCGTGTCTC |
|  |  |  |
| NEFL | TAAATAGGGGTGGCAGAACG | AGAGGGAAGGGGGAGGAT |
|  |  |  |
| Nestin | TTAATGGCCAGGGTCCCAAC | GCTCCAGCCCGTTCATCACT |
|  |  |  |
| NeuN | GCGGCTACACGTCTCCAACATC | ATCGTCCCATTCAGCTTCTCCC |
|  |  |  |
| NKX6-1 | GCCTCGGAGAACGAGGAAGA | CGCTGCTGGACTTGTGCTTC |
|  |  |  |
| NURR1 | TATTCCAGGTTCCAGGCGAA | GCTAATCGAAGGACAAACAG |
|  |  |  |
| Pax6 | CCAGGGCAATCGGTGGTAGT | ACGGGCACTCCCGCTTATAC |
|  |  |  |
| S100b | GGAGACGGCGAATGTGACTT | GAACTCGTGGCAGGCAGTAGTAA |
|  |  |  |
| Sox1 | CAGCAGTGTCGCTCCAATTCA | GCCAAGCACCGAATTCACAG |
|  |  |  |
| Sox 2 | TGGCGAACCATCTCTGTGGT | CCAACGGTGTCAACCTGCAT |
|  |  |  |
| Syt1 | TCATTCAGCTTTGAAGTACCTTT | GCATCGTTCTTGCCAATCTT |
|  |  |  |
| TBR2 | CCCAGACCCAACCTTTCC | GAGCCAATTTCCTCTTTCACTT |
|  |  |  |
| TH | TGTCTGAGGAGCCTGAGATTCG | GCTTGTCCTTGGCGTCACTG |
|  |  |  |
| TUBB3 | CGCCCTCCTGCAGTATTTATGG | TCAGGCCTGGAGCTGCAATA |
